# Sociodemographic Disparities in the Relationship between Living Alone and Suicide Ideation among Older Adults

**DOI:** 10.1007/s11126-025-10169-z

**Published:** 2025-06-13

**Authors:** Ping-I Lin, James R. John, George Grossberg, Jeffrey F. Scherrer, Erick Messias

**Affiliations:** 1 Discipline of Psychiatry and Mental Health, University of New South Wales, Sydney, NSW Australia; 2 Department of Psychiatry and Behavioral Neuroscience, School of Medicine, Saint Louis University, 1438 S Grand Blvd, St. Louis, MO 63104 USA; 3 School of Medicine, Western Sydney University, Sydney, NSW Australia; 4 Department of Family and Community Medicine, School of Medicine, Saint Louis University, St. Louis, MO USA

**Keywords:** Suicide, Living arrangement, Gender, Race, Household income

## Abstract

This study aimed to investigate the relationship between living arrangements and suicide ideation, while examining how sociodemographic factors, including race and income, interact with living arrangements to influence the likelihood of these behaviors. Data on 149,996 individuals aged 50 and above were extracted from the National Surveys on Drug Use and Health (NSDUH) from 2020 to 2022, a nationally representative, annual, cross-sectional survey of U.S. residents. The study focused on individuals aged 50 and above in three racial groups (White individuals, Black individuals, and Hispanic individuals) and utilized multiple logistic regression models to analyze the data. The results show that living alone was not a significant predictor for suicide ideation after adjusting for depression, and gender, but the association between living alone and suicide ideation was moderated by race and income. Specifically, the association between living alone and suicide ideation was stronger in Black individuals compared to White individuals (AOR = 2.70, 95% CI: 1.06–6.87, *p* = 0.037). Additionally, the association between living alone and suicide ideation was smaller in the third income category (annual income $50,000 - $74,999) than first income category (annual income < $20,000) (AOR = 0.29, 95% CI: 0.09–0.94, *p* = 0.039). These findings underscore the importance of targeted interventions to reduce suicidal behaviors in vulnerable populations.

**Supplementary Information:**

The online version contains supplementary material available at 10.1007/s11126-025-10169-z.

## Paper highlights

Using nationally representative data from NSDUH, this study examined how living arrangements and sociodemographic factors influence suicide ideation in older adults.

While living alone was not a direct predictor of suicide ideation after adjusting for depression and sociodemographic factors, its impact could vary by race and income, with certain racial groups experiencing stronger associations and higher income categories mitigating risk.

The findings underscore the need for tailored suicide prevention strategies that address social isolation and sociodemographic vulnerabilities in older populations.

**Supplementary Information:**

The online version contains supplementary material available at 10.1007/s11126-025-10169-z.

## Introduction

The increase in suicide rates has been found to be more pronounced in older individuals than younger individuals in recent years in the US, with the largest percent increase in suicide rates between 2001 and 2021 among men ages 55–64 and among women ages 65–74 [1]. This trend underscores the urgent need to better understand the factors contributing to suicide risk in older adults, who may face unique challenges such as social isolation, chronic health conditions, and diminished access to social and community support. Factors such as living arrangement, socioeconomic status, and social support have been shown to significantly influence suicide risk among older adults [2, 3]. Specifically, accumulating evidence demonstrates that living alone is associated with a higher risk of suicide among older individuals [4–9]. Therefore, the relationship between living alone and suicidality may provide valuable insights into the factors influencing suicide risk among older individuals.

The proportion of adults aged 50–80 reporting loneliness is estimated to be 37–41% from 2021 to 2024 [10]. The link between social isolation and suicide risk has been well documented across various populations [11, 12]. Living alone is commonly used as an objective indicator of social isolation, which does not necessarily reflect individuals’ subjective experiences of loneliness or perceived social disconnection. Prior research has shown that loneliness—defined as the distressing experience that occurs when one’s social relationships are perceived as inadequate—may be a more proximal and potent predictor of suicidal thoughts and behaviors in older adults than structural living arrangements alone. Additionally, 

although living alone is not synonymous with social isolation or loneliness, it may lead to reduced immediate social support [13]. Living with a partner may be associated with a lower risk of self-harm hospitalization associated with loneliness [12]. Elderly individuals living alone also tend to have higher levels of suicidal ideation, depression, and hopelessness, and lower self-esteem compared to those living with family [14]. However, family connectedness can moderate this risk, with stronger family connections reducing the likelihood of suicide ideation [15]. However, a recent study based on the data from 50-state COVID-19 surveys does not endorse living alone to be associated with suicide ideation among elderly individuals [16]. The lack of association between living alone and suicide risk may arise from heterogeneity related to sociodemographic features.

While existing research highlights the multifaceted relationship between living alone and suicide risk among older adults, the complexities of this association require deeper investigation. Previous evidence suggests sociodemographic factors such as income may buffer the psychological impact of living alone for older individuals [17]. Second, research indicates that racial/ethnic background significantly influences mental health-seeking behaviors, particularly among older adults. Studies show that older adults from ethnic/racial minority groups are less likely to use mental health services compared to white counterparts [18, 19]. Factors such as language barriers, stigma, and lack of culturally appropriate care contribute to these disparities [20, 21].

Understanding how sociodemographic factors interact with living alone to jointly correlate with suicide ideation is critical for identifying key risk factors and potential intervention points for suicide prevention in older individuals. Focusing on suicide ideation, rather than suicide deaths, allows for the identification of individuals at an earlier stage of the suicidal process, providing opportunities for timely intervention. Suicide ideation is often a precursor to suicide attempts and deaths, making it a valuable indicator for assessing underlying risk factors and developing preventative strategies [22]. Moreover, studying suicide ideation offers a broader scope of analysis, as ideation is more prevalent than completed suicides, providing a larger sample size and greater statistical power to detect significant patterns and interactions. This is particularly important when examining subgroups, such as older adults living alone, where the number of suicide deaths may be too small to yield reliable conclusions. The results of this research may pave the way for developing targeted and effective strategies to reduce suicidal behaviors among older adults, addressing specific vulnerabilities related to social isolation and demographic disparities. Therefore, the objectives of this study are as follows:

- To investigate the relationship between living arrangements (living alone versus living with others) and suicide ideation in older adults.
- To examine how sociodemographic factors, including race and income, interact with living arrangements to influence the likelihood of suicide ideation.

By addressing these objectives, the study aims to provide actionable insights to guide policy development and clinical practices, ultimately contributing to the prevention of suicidal behaviors and improving the mental health of older adults.

## Methods

### Study Design and Data Source

The data were extracted from the National Surveys on Drug Use and Health (NSDUH) data, a nationally representative, annual, cross-sectional survey employing a multistage probability sampling strategy. This ensures a diverse and representative sample of US residents aged 12 years and above, capturing variations across demographic and geographic subgroups. We combined data from the three-year period of 2020 to 2022, ensuring robust sample sizes and greater statistical power to examine subgroup differences. It provides nationally representative estimates and comprehensive sociodemographic, health, and living arrangement variables, enabling us to investigate the relationship between living alone and health-related outcomes while adjusting for confounders. Its inclusion of a wide range of substance use, mental health, and social behavior variables further supports the nuanced exploration of the research question. Before merging the data, we evaluated whether there were significant differences in the proportion of individuals living alone across the three years to confirm the appropriateness of pooling the data for analysis.

### Key Variables

The outcome variables of interest comprised of three measures related to suicide and self-harm which included suicidal ideation (yes/no), suicide plan (yes/no), and suicide attempt (yes/no) in the past 12 months. The primary exposure in this study was the living arrangement at the time of reporting which was dichotomized as living alone versus living with others. We also included other key covariates such as age, gender, race, annual income, depression in the past 12 months (yes, no), and alcohol use in the past 12 months. The proportion of individuals living alone exceeded 10% for individuals in the age group of 50 years and above. Similarly, White individuals (European-descent individuals), Black individuals (African-descent individuals), and Hispanic individuals are the three racial groups with at least 10% of the respondents living alone. Therefore, the subsequent analyses are focused on individuals aged 50 and above in these three racial groups.

### Statistical Analysis

Sampling weights were applied for all analyses to manage sampling error and for non-responses. Further details on sample weights can be found in the Substance Abuse and Mental Health Services Administration (SAMHSA) report. 79.4% of the participants had non-missing data on suicide ideation. Listwise deletion was adopted to main the integrity of the dataset.

We utilized descriptive statistics to identify the sociodemographic characteristics associated with suicidal/self-harm behaviors of the sample using Pearson’s chi-squared tests. To assess whether the relationship between living alone and suicide ideation varied by sociodemographic characteristics, we used generalized linear models with interaction terms to test for effect modification by race and income. These interactions were examined in two separate models to preserve statistical power and ensure stable estimates. All analyses were conducted using Stata’s survey (svy) procedures, which account for the complex sampling design of the NSDUH, including clustering, stratification, and unequal probabilities of selection. This approach ensures that standard errors, confidence intervals, and p-values are adjusted for design effects, thereby improving the accuracy and generalizability of the findings. Both models accounted for depression and gender as covariates. The marginal effect of each predictor was reported as adjusted odds ratios (AOR). All statistical analyses were performed using the software STATA 18 [23].

## Results

The characteristics of 27,818 adults stratified by living arrangements are presented in Table 1. There was no statistically significant difference in the proportion of individuals living alone across the three consecutive years, and hence we merged the data from these three years’ surveys. In terms of age, those aged 50–64 years constituted a larger proportion of individuals living alone, while those aged 65 years and older were more prevalent among those living with others (*p* < 0.001). Gender distribution also differed significantly, with a higher percentage of females living alone compared to males, while males were more common among those living with others (*p* < 0.001). Regarding race, a higher percentage of Black individuals resided alone compared to those living with others, whereas White individuals were more evenly distributed across living arrangements (*p* < 0.001). Income disparities were evident (*p* < 0.001), with a larger proportion of individuals earning less than $20,000 per year living alone, whereas those with higher incomes were more likely to live with others. Concerning mental health indicators, higher rates of suicidal ideation, suicidal plan, suicide ideation, and depression were observed among individuals living alone, while alcohol use was slightly higher among those living with others (*p* < 0.010).

**Table 1:** Characteristics of the sample stratified by living arrangements. The percentage within each subgroup (i.e., living alone and living with others, separately) is presented

Characteristics	Total (*N* = 27818) Weighted sample (*N* = 352798004) %	Living alone (*N* = 6535) Weighted sample (*N* = 76620911) %	Living with others (*N* = 21283) Weighted sample (*N* = 276177093) %	*p*-value
Time period				0.154
2020	32.9	31.7	33.3	
2021	33.5	35.1	33.0	
2022	33.6	33.2	33.8	
Age				< 0.001
50–64 years	52.5	42.7	55.3	
65 years and older	47.5	57.3	44.7	
Gender				< 0.001
Male	47.4	43.2	48.6	
Female	52.6	56.8	51.4	
Race				< 0.001
White	61.2	69.0	60.0	
Black	12.2	14.9	11.8	
Hispanic	17.8	19.0	9.8	
Income				< 0.001
Level 1: Less than $20,000	12.8	29.1	7.8	
Level 2: $20,000 - $49,999	26.8	36.2	23.9	
Level 3: $50,000 - $74,999	16.4	15.1	16.8	
Level 4: $75,000 and above	44.0	19.5	51.6	
Suicide ideation– past 12 months				< 0.001
No	95.0	96.8	98.0	
Yes	2.2	3.2	2.0	
Missing/Undisclosed	2.8			
Depression– past 12 months				< 0.001
No	85.6	85.3	89.4	
Yes	11.2	14.7	10.6	
Missing/Undisclosed	3.2			

* Suicidality is defined as the presence of suicidal ideation, suicide plan, or suicide attempt

Two multiple logistic models were used to examine the associations between living arrangements, income, race, and their interactions on suicide ideation. Including multiple interaction terms in the same model can result in smaller subgroups, particularly when analyzing complex stratifications of categorical variables. This can lead to imprecise estimates, wider confidence intervals, and potential type II errors (i.e., failure to detect significant effects when they exist) [24]. Additionally, including both interaction terms (i.e., Living Alone × Race and Living Alone × Income) may introduce multicollinearity. Using two separate multiple logistic models to evaluate the interaction effects between living alone and the two sociodemographic factors (i.e., race and income) allows for a more robust analysis by avoiding the limitations associated with sample size reductions when multiple interaction terms are included in a single model. This approach helps ensure statistical power and reduces the risk of overfitting, which is particularly important in survey-based datasets or when examining relatively rare outcomes, such as suicide ideation.

In the model evaluating the interaction between living arrangements and race, living alone was not significantly associated with suicide ideation overall (AOR = 1.04, 95% CI: 0.74–1.47, *p* = 0. 804). Black individuals living alone had a significantly higher likelihood of suicide ideation compared to White individuals (AOR = 2.70, 95% CI: 1.06–6.87, *p* = 0.037). No significant interaction effect was found for Hispanic individuals living alone compared to White individuals (AOR = 0.85, 95% CI: 0.23–3.15, *p* = 0.804).

**Table 2:** Multivariable logistic regression models with suicide ideation as the outcome and a focus on interaction between living arrangement and race

Predictor	AOR	95% CI	*p*-value
Living alone	1.04	0.74–1.47	0.804
Living alone x Race (compared with White individuals not living alone)			
Black individuals living alone	2.7	1.06–6.87	0.037
Hispanic individuals living alone	0.85	0.23–3.15	0.804
Depression	16.41	12.30–21.89	< 0.001
Gender (Male vs. Female)	0.74	0.56–0.98	0.033
Income			
Level 2 vs. Level 1	0.77	0.52–1.16	0.209
Level 3 vs. Level 1	0.75	0.47–1.21	0.245
Level 4 vs. Level 1	0.36	0.24–0.55	< 0.001
Race			
Black individuals vs. White individuals	0.49	0.28–0.87	0.015
Hispanic individuals vs. White individuals	0.77	0.42–1.41	0.398

Income level coded as 1: < $20,000, 2: $20,000– $49,999, 3: $50,000 - $74,999, 4: $75,000 and above

**Fig. 1:**
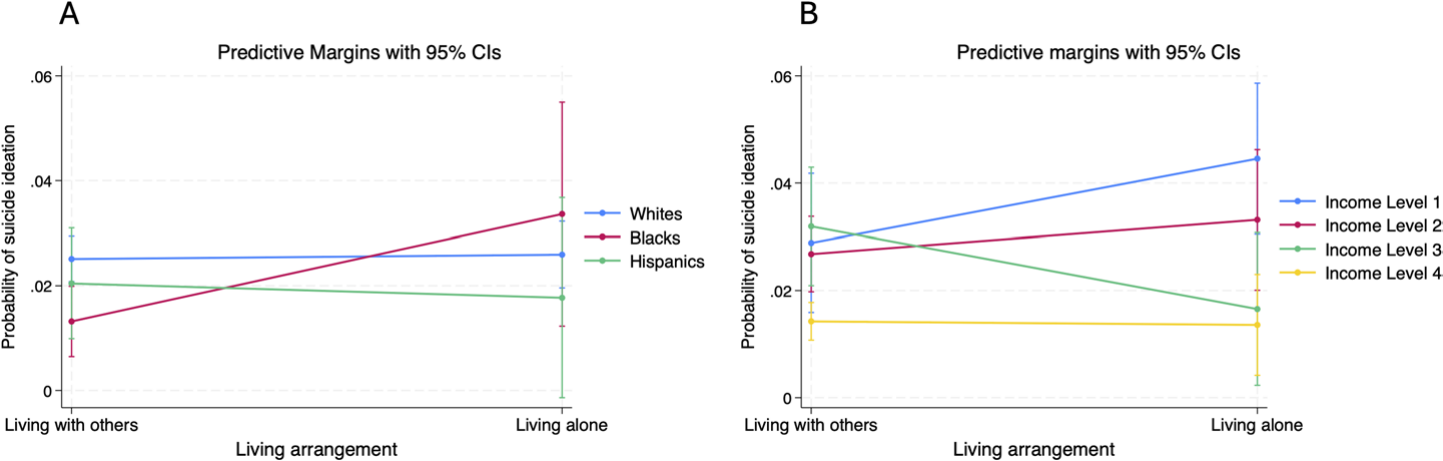
Risk of suicide ideation stratified by living arrangement and sociodemographic factors. Panel A and Panel B present the predicted probability of suicide ideation stratified by race and income, respectively. Income level coded as 1: < $20,000, 2: $20,000– $49,999, 3: $50,000 - $74,999, 4: $75,000 and above

In the model evaluating the interaction between living arrangements and income, living alone was also not significantly associated with suicide ideation overall (AOR = 1.63, 95% CI: 0.89–2.98, *p* = 0.114). However, among individuals in the third income category (i.e., $50,000–$74,999), living alone was associated with a significantly lower likelihood of suicide ideation than individuals in the first income category (i.e., less than $20,000) (AOR = 0.29, 95% CI: 0.09–0.94, *p* = 0.039). No significant interaction effects were observed for the other income categories (*p* > 0.05).

Depression was a strong and consistent predictor of suicide ideation in both models, with an AOR of 16.41 (95% CI: 12.30–21.89, *p* < 0.001). Gender was also significantly associated with suicide ideation, with females having a lower likelihood compared to males (AOR = 0.74, 95% CI: 0.56–0.98, *p* = 0.033). Both models demonstrated acceptable fit, as indicated by pseudo-R^2^ values of 0.21 and 0.23, respectively, which suggest that approximately 21% and 23% of the variance in suicide ideation is explained by the models.

**Table 3:** Multivariable logistic regression models with suicide ideation as the outcome and a focus on interaction between living arrangement and income level

Predictor	AOR	95% CI	*p*-value
Living alone	1.63	0.89–2.98	0.114
Living alone X Income (compared with individuals with a level 1 income and living with others)			
Level 2 vs. Level 1	0.75	0.34–1.68	0.487
Level 3 vs. Level 1	0.29	0.09–0.94	0.039
Level 4 vs. Level 1	0.72	0.27–1.91	0.509
Depression	16.41	12.30-21.89	< 0.001
Gender (Male vs. Female)	0.74	0.56–0.98	0.033
Income*			
Level 2 vs. Level 1	0.93	0.53–1.64	0.807
Level 3 vs. Level 1	1.11	0.59–2.08	0.754
Level 4 vs. Level 1	0.45	0.26–0.79	0.006
Race			
Black individuals vs. White individuals	0.79	0.49–1.31	0.356
Hispanic individuals vs. White individuals	0.77	0.45–1.33	0.355

Income level coded as 1: < $20,000, 2: $20,000– $49,999, 3: $50,000 - $74,999, 4: $75,000 and above

## Discussion

### What the Current Study Adds

Our study’s findings shed novel insights into the relationship between living alone and suicide ideation among older adults in the context of racial differences. While the overall risk of suicide ideation is lower in Black older individuals compared to their White counterparts, the impact of living alone appears significantly more pronounced in Black older individuals. This suggests that living alone may exacerbate feelings of isolation or lack of support in this demographic group, leading to increased vulnerability to suicidal ideation or behavior. This finding has lent some support to previous research indicating that Black individuals with mental health issues are more likely to experience distress stemming from chronic exposure to socioeconomic disadvantage and racial discrimination [25]. However, such a finding contradicts with the study by Olfson et al. that found that the relationship between living alone and suicide deaths was lowest in Black individuals [4]. The difference might be at least partially attributable to different suicidal outcomes (i.e., suicide ideation versus suicide deaths) and the unique impact of the COVID-19 impact since the study by Olfson et al. and the current study were conducted before and during the pandemic, respectively. The COVID-19 pandemic disproportionately affected Black Americans compared to their white counterparts, with higher rates of infection, hospitalization, and mortality [26–28]. Additionally, Black Americans are more likely to live in crowded urban areas and face economic challenges, such as job losses and food insecurity, which can exacerbate the pandemic’s impact. The combination of these socioeconomic factors, along with systemic racism and health inequities, impacted Black individuals to a greater extent than White individuals in the U.S., especially those living alone [29, 30].

Further, a growing body of research indicates that Black Americans experience elevated levels of stigma and discrimination, which significantly affect their psychological well-being [31–33]. Our findings hence highlight the necessity for tailored interventions that not only address mental health concerns but also consider the contextual influence of living arrangements and racial factors. Such a perspective shifts the narrative from viewing living alone as a universal risk factor for suicide ideation to recognizing its differential impact based on individual characteristics and avoiding oversimplification for suicide prevention in older individuals [34].

Our findings that income moderates the relationship between living alone and suicide ideation align with growing evidence that older adults living in poverty face increased risks of mortality and morbidity due to either physical or psychiatric conditions [35–39]. Although the study found an inverse association between income and suicide ideation, the moderating effect of income on the link between living alone and suicide ideation did not follow a consistent dose-response pattern. Our findings also contrast the finding from the study by Olfson et al., which found that the suicide death rate was greatest along those living alone with annual incomes greater than $125,000 [4]. One meta-analysis of 27 studies found mixed results for the relationship between income inequality and psychological wellbeing across various populations. Some studies found that income inequality within communities exacerbates mental health issues, particularly among individuals in lower socioeconomic strata who may feel marginalized or excluded. Conversely, other studies suggested that individual income, rather than societal inequality, plays a stronger role in determining mental health outcomes, underscoring the importance of context [40]. While lower income levels are often associated with increased psychological distress and greater suicide risk due to financial strain, reduced access to healthcare, and social isolation, this relationship is not always straightforward. Higher income levels may not always serve as a protective factor, as they can introduce their own stressors, such as pressure to maintain status, workplace demands, or the potential for reduced social support in highly affluent communities [41]. Therefore, the relationship between income levels and suicidal outcomes may extend beyond the association between financial capacity and psychological wellbeing.

### Strengths and Limitations

The study draws data from the NSDUH, ensuring a nationally representative sample of US residents. By combining data from three consecutive years, the study enhances statistical power and sample size, thereby improving the robustness of the analysis. Comprehensive adjustment for key covariates in the multivariable logistic regression analysis enhances the validity of results by minimizing confounding effects. Additionally, the utilization of sampling weights and consideration of non-responses in the statistical analysis improve the accuracy of estimates, reducing biases associated with survey data.

This study faces several methodological limitations. First, its cross-sectional design limits causal inference between living arrangements and suicide ideation. Second, reliance on self-reported measures may introduce bias, and unmeasured confounders like social support and physical health status could affect results. Third, interaction terms were included but may not fully capture the complexity of sociodemographic factors, and potential mediating pathways were not explored. Fourth, generalizability is limited to the US context, and broad racial and income categorizations may obscure important variations. Additionally, the age groupings used might not capture the full diversity of experiences within the older adult population. Addressing these limitations in future research could enhance understanding and inform targeted interventions.

## Conclusions

By examining how sociodemographic factors intersect with living arrangements to influence suicide risk, we can develop targeted interventions and support systems that address the unique needs of vulnerable populations. Understanding these interactions allows for the creation of evidence-based policies and community programs to address health inequality to mitigate social isolation and its psychological impact on older adults. Such research not only deepens our understanding of suicide ideation in this demographic but also informs tailored prevention strategies, ultimately reducing suicide risk and promoting mental well-being among older individuals.

Further, as part of routine mental health assessments in older adults, we recommend incorporating validated tools to assess subjective social isolation, such as the UCLA Loneliness Scale [42], alongside objective measures like living arrangements. This dual approach can help clinicians more accurately identify individuals at elevated risk for suicide ideation by capturing both structural and perceived dimensions of social disconnection. More importantly, suicide prevention strategies need to consider the intersecting effects of race, income, and living arrangements. Tailored interventions that address the unique social and economic contexts of older adults may be more effective in mitigating suicide risk in high-vulnerability groups.

## Electronic Supplementary Material

Below is the link to the electronic supplementary material.

Supplementary Material 1

## Data Availability

The data used in this study were obtained from the National Surveys on Drug Use and Health (NSDUH), a publicly available dataset provided by the Substance Abuse and Mental Health Services Administration (SAMHSA). The NSDUH data can be accessed through the SAMHSA website or the Inter-university Consortium for Political and Social Research (ICPSR) repository. Researchers interested in using this dataset can obtain it following the access guidelines outlined by SAMHSA. No additional data were generated or collected specifically for this study.
