# Supplementary material for: Sociodemographic Disparities in the Relationship between Living Alone and Suicide Ideation among Older Adults
Source: Psychiatr Q. 2025 Jun 13;97(1):203–14. doi: 10.1007/s11126-025-10169-z (PMC13032931; doi:10.1007/s11126-025-10169-z)
Supplement: Supplementary file 1 — Supplementary Material 1 [file 11126_2025_10169_MOESM1_ESM.docx]

**Disclosure of Interest**

The authors have no conflicts of interest to declare.
